# A gut microbiome‐lipid axis in early pregnancy is associated with metabolic dysregulation and diabetes risk

**DOI:** 10.1002/imt2.70166

**Published:** 2026-08-10

**Authors:** Zhonghan Sun, Chang He, Xiaonan Ma, Ping Wu, Tianlei Wang, Jiaying Yuan, Yanni Pu, Xiaofeng Zhou, Zhendong Mei, Huiling Song, Yetong Wang, Haiyan Yue, Yuanqing Fu, Jusheng Zheng, An Pan, Da Chen, Shangyu Hong, Xiong‐Fei Pan, Yan Zheng

**Affiliations:** ^1^ State Key Laboratory of Genetics and Development of Complex Phenotypes, School of Life Sciences Shanghai Institute of Infectious Disease and Biosecurity, Zhongshan Hospital Shanghai China; ^2^ State Key Laboratory of Genetics and Development of Complex Phenotypes, School of Life Sciences Human Phenome Institute, Zhongshan Hospital Shanghai China; ^3^ Department of Epidemiology and Biostatistics, Ministry of Education Key Laboratory of Environment and Health, School of Public Health, Tongji Medical College Huazhong University of Science and Technology Wuhan China; ^4^ Laboratory of Epidemiology and Population Health & Children's Medicine Key Laboratory of Sichuan Province, West China Institute of Women and Children's Health West China Second University Hospital, Sichuan University Chengdu China; ^5^ Department of Obstetrics and Gynecology West China Second University Hospital, Sichuan University Chengdu China; ^6^ Key Laboratory of Birth Defects and Related Diseases of Women and Children Sichuan University Chengdu China; ^7^ Shuangliu Institute of Women's and Children's Health Shuangliu Maternal and Child Health Hospital Chengdu China; ^8^ Department of Science and Education Shuangliu Maternal and Child Health Hospital Chengdu China; ^9^ Department of Medicine Brigham and Women's Hospital and Harvard Medical School Boston USA; ^10^ Affiliated Hangzhou First People's Hospital, School of Medicine Westlake University Hangzhou China; ^11^ College of Environment and Climate, Guangdong Key Laboratory of Environmental Pollution and Health Jinan University Guangzhou China; ^12^ State Key Laboratory of Genetics and Development of Complex Phenotypes, Department of Endocrinology, Shanghai Pudong Hospital School of Life Sciences, Fudan University Pudong Medical Center, Fudan University Shanghai China; ^13^ Ministry of Education Key Laboratory of Public Health Safety, School of Public Health, Institute of Nutrition Fudan University Shanghai China; ^14^ National Clinical Research Center for Aging and Medicine, Huashan Hospital Fudan University Shanghai China

**Keywords:** birth cohort, gestational diabetes mellitus, gut microbiome, lipidome, lipid/glucose metabolism

## Abstract

Gestational diabetes mellitus (GDM) reflects metabolic dysregulation that becomes clinically apparent during pregnancy and shares key pathophysiological features with broader forms of diabetes. Gut microbiome‐host metabolic interactions may contribute to this process, yet their role in early pregnancy remains incompletely understood. In this prospective nested case‐control study within the Tongji‐Huaxi‐Shuangliu Birth Cohort, 784 pregnant women, including 222 who developed GDM, underwent first‐trimester gut metagenomic and plasma lipidomic profiling. Cross‐omics analyses were performed to identify microbiome‐lipid associations and potential mediation patterns. Women who later developed GDM showed reduced gut microbial diversity and altered microbial profiles in early pregnancy. We identified 26 microbial species associated with GDM risk, with seven species, including *Ruminococcus bicirculans* (*R. bicirculans*), showing concordant associations in external type 2 diabetes populations. Microbial pathways related to fatty acid and lipid biosynthesis were enriched in women at higher risk. Plasma lipidomics revealed widespread alterations, particularly among glycosphingolipid‐related metabolites. Integrated analyses suggested that lipidomic variation statistically accounted for part of the microbiome‐GDM association. A class‐level dihexosylceramide feature, DHC 24:1, consistent with lactosylceramide‐related metabolites, emerged as a potential mediator and was prioritized for exploratory follow‐up. Experimental analyses provided functional support for a microbiome‐lipid‐host interaction axis. *R. bicirculans* promoted lactosylceramide 24:1 production *in vitro*, bacterial colonization and metabolite administration improved insulin tolerance *in vivo*, and lactosylceramide 24:1 modulated insulin‐stimulated AKT signaling dynamics in hepatocytes. These findings identify a gut microbiome‐lipid axis associated with metabolic dysregulation in pregnancy and suggest a potential mechanism linking microbial metabolism to host insulin signaling.

## Highlights

- First‐trimester microbiome and lipidome profiles revealed early molecular signatures preceding gestational diabetes mellitus (GDM) diagnosis.
- Integrated analyses revealed a microbiome‐glycosphingolipid axis, with dihexosylceramide (DHC)/lactosylceramide (LacCer) 24:1 linking *Ruminococcus bicirculans* to GDM‐related metabolic dysregulation.
- Functional follow‐up experiments showed that *R. bicirculans* colonization and LacCer 24:1 administration improved insulin tolerance and that LacCer 24:1 modulated hepatic AKT signaling.
- Combining microbial and lipidomic risk scores identified women with markedly elevated GDM risk in early pregnancy.

## Graphical abstract

Early‐pregnancy microbiome and lipidome profiling identified a microbiome‐glycosphingolipid axis associated with later gestational diabetes mellitus (GDM) risk. In the prospective pregnancy cohort, women who later developed GDM showed altered gut microbial composition, enriched microbial lipid biosynthesis pathways, and broad plasma lipidomic changes before clinical diagnosis. Cross‐omics analyses highlighted dihexosylceramide (DHC) 24:1 as a lipid feature statistically linking GDM‐associated microbial signatures with metabolic dysregulation. Experimental evaluation further supported the biological plausibility of this axis, showing that *Ruminococcus bicirculans* promoted lactosylceramide (LacCer) 24:1 production and LacCer‐related metabolism may modulate host insulin signaling.

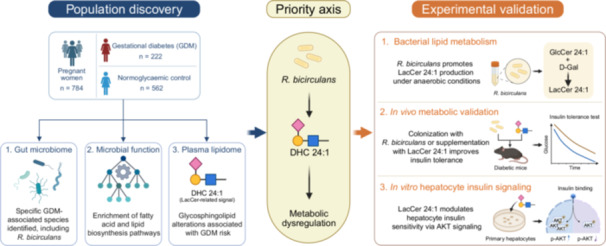

## INTRODUCTION

Gestational diabetes mellitus (GDM) is a common metabolic complication of pregnancy and is associated with substantial short‐ and long‐term health consequences for both mothers and offspring [1, 2]. Women with a history of GDM are at a 10‐fold increased risk of type 2 diabetes mellitus (T2DM), while their offspring are more susceptible to metabolic disorders later in life [3, 4]. As GDM is typically diagnosed in mid‐pregnancy, current screening strategies may miss earlier biological changes that precede overt hyperglycemia, limiting opportunities for early intervention [5]. Conventional early‐pregnancy risk assessment based on maternal age, body mass index (BMI), and family history also has limited discriminatory ability [6]. A better understanding of the biological processes that underlie early metabolic maladaptation in pregnancy may therefore improve both mechanistic insight and early risk stratification.

GDM arises from the interaction of genetic susceptibility and environmental exposures, including diet and physical activity, against the background of profound metabolic adaptation during pregnancy [1]. Increasing evidence indicates that both the gut microbiome and circulating metabolites are altered in GDM [7, 8, 9, 10]. In particular, early‐pregnancy lipidomic profiles have been associated with subsequent GDM risk [8], and the gut microbiome has emerged as an important regulator of host lipid metabolism and broader cardiometabolic phenotypes [11, 12, 13, 14, 15]. However, how gut microbial alterations are translated into host metabolic phenotypes through defined molecular intermediates remains largely unclear. In particular, whether specific lipid metabolites act as mediators linking gut microbiota to host metabolic dysregulation in early pregnancy has not been well established.

**Figure 1:**
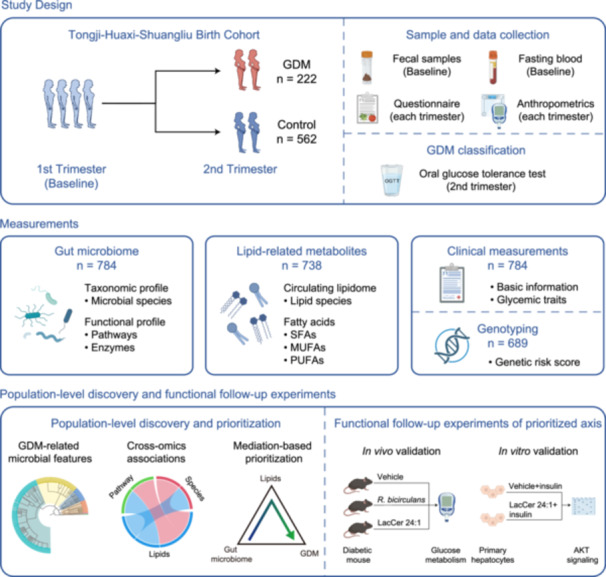
Study design, multi‐omics measurements, and analytical framework. Pregnant women from the Tongji‐Huaxi‐Shuangliu Birth Cohort were enrolled in the first trimester and followed to ascertain gestational diabetes mellitus (GDM) in the second trimester. At baseline (first trimester), fecal samples were collected for shotgun metagenomic profiling, blood samples for plasma lipidomic and genetic analyses, and clinical phenotypes were recorded. The multi‐omics measurements included gut microbial taxonomic and functional profiling, circulating lipid and fatty acid profiling, clinical phenotyping, and genetic data. Population‐level analyses were performed to identify GDM‐related microbial features, characterize cross‐omics associations, and prioritize candidate microbiome‐lipid axes through mediation analysis. Functional follow‐up experiments then focused on the prioritized *Ruminococcus bicirculans*‐glycosphingolipid axis using *in vivo* and *in vitro* models. The schematic was created with BioRender.com. AKT, protein kinase B; GDM, gestational diabetes mellitus; LacCer, lactosylceramide; MUFAs, monounsaturated fatty acids; OGTT, oral glucose tolerance test; PUFA, polyunsaturated fatty acid; *R. bicirculans*, *Ruminococcus bicirculans*; SFAs, saturated fatty acids.

In this prospective nested case‐control study of 784 pregnant women, we performed integrated profiling of the first‐trimester gut microbiome and plasma lipidome, together with detailed clinical phenotyping (Figure 1). We aimed to identify microbial taxa and functional pathways associated with later GDM, to characterize their relationships with circulating lipid metabolites, and to define candidate microbiome‐lipid interaction axes for experimental validation. Given the shared pathophysiology between GDM and broader forms of diabetes, we further examined the generalizability of key microbial signatures in T2DM populations and evaluated whether multi‐omics integration could refine early risk stratification.

## RESULTS

### Characteristics of the study population

This study included 784 pregnant women (mean age ± standard deviation: 27.7 ± 3.9 years) who provided fecal samples during the first trimester (T1), including 222 who developed GDM and 562 who maintained glycemic homeostasis throughout pregnancy (see Methods) (Figure 1). Compared with women who remained normoglycemic, those who later developed GDM already exhibited a less favorable metabolic profile in early pregnancy, with higher adiposity, blood pressure, glycemic markers, and selected lipid‐related traits at baseline (all *p* < 0.05, Table S1). Although fasting glucose and glycated hemoglobin A1c (HbA1c) decreased from the first to the second trimester in both groups, these reductions were attenuated in women who later developed GDM (both *p* < 0.05, Table S1). By contrast, age, gestational age, parity, education level, smoking and drinking history, and family history of T2DM were broadly similar between groups (all *p* > 0.05, Table S1). These findings indicate that metabolic differences were already detectable in early pregnancy, before clinical diagnosis of GDM.

### Alterations of the early‐pregnancy gut microbiome were associated with later GDM development

**Figure 2:**
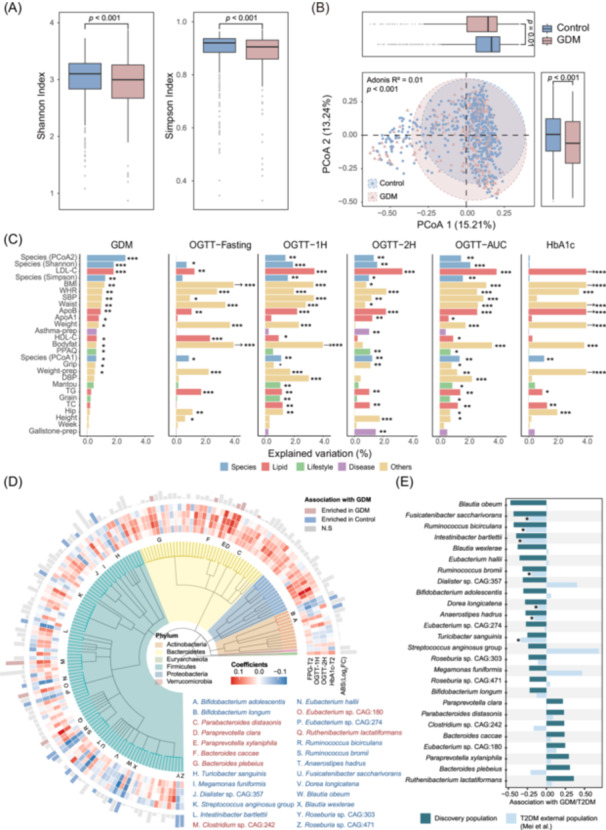
Association of the early‐pregnancy gut microbiome with later GDM. (A) Species‐level α‐diversity of the gut microbiome in women who later developed GDM and those who remained normoglycemic, represented by the Shannon and Gini‐Simpson indices. (B) Principal coordinate analysis of gut microbial composition based on Bray‐Curtis distance at the species level. (C) Proportion of variance in GDM status and second‐trimester glycemic traits explained by baseline gut microbial composition, lipid‐related traits, and selected clinical factors *p < 0.05; **p < 0.01; ***p < 0.001. (D) Phylogenetic representation of microbial species associated with later GDM (FDR < 0.05). Outer rings indicate associations of these species with second‐trimester glycemic traits and GDM status (red, positive association; blue, inverse association; bar height, absolute value of log_2_‐transformed fold change). Models were adjusted for maternal age, gestational age, education, parity, family history of diabetes, history of PCOS, pre‐pregnancy BMI, cigarette smoking, alcohol drinking, physical activity, and antibiotic use. (E) Comparison of associations for selected microbial species in the current GDM cohort and an external T2DM dataset (Mei et al. [16]), with bar length representing regression coefficients estimated by MaAsLin2. The external analysis was adjusted for age, sex, and BMI. Asterisks indicate statistically significant associations in both datasets with concordant direction. Unless otherwise stated, models were adjusted for maternal age, gestational age, pre‐pregnancy BMI, smoking, alcohol drinking, education, parity, family history of diabetes, physical activity, and antibiotic use. ApoA1, apolipoprotein A1; ApoB, apolipoprotein B; BMI, body mass index; DBP, diastolic blood pressure; FDR, false discovery rate; FPG, fasting plasma glucose; HbA1c, glycated hemoglobin A1c; LDL‐C, low‐density lipoprotein cholesterol; N.S., not significant; PCoA, principal coordinate analysis; PPAQ, Pregnancy Physical Activity Questionnaire; SBP, systolic blood pressure; T2DM, type 2 diabetes mellitus; TC, total cholesterol; TG, triglyceride; WHR, waist‐to‐hip ratio.

At baseline (T1), all 784 participants provided fecal samples for whole‐genome shotgun metagenomic sequencing, yielding an average of 58.7 million high‐quality reads per sample (~8.5 Gb). After quality control and filtering, 163 microbial species and 327 functional pathways were retained for downstream analyses (Tables S2 and S3). Compared with women who remained normoglycemic throughout pregnancy, those who later developed GDM exhibited lower species‐level α‐diversity in early pregnancy, as reflected by both the Shannon and Gini‐Simpson indices (both *p* < 0.01, Figure 2A). Although the overall gut microbial community structure showed substantial overlap between groups, permutational multivariate analysis of variance (PERMANOVA) identified a modest but significant difference in beta diversity between GDM and control participants (Adonis *R* ^2^ = 0.01, *p* < 0.001, Figure 2B), suggesting shifts in overall microbiome composition associated with GDM.

We next examined whether early‐pregnancy gut microbial features were related to later metabolic phenotypes. Among baseline host factors and microbial measurements, overall microbial composition explained a greater proportion of variance in GDM status than conventional clinical variables, including LDL‐C, while microbial diversity also showed a significant contribution (Figure 2C). In addition, baseline microbial composition explained more variance in second‐trimester OGTT‐derived glucose traits (range from 1.2% to 2.0%) than in HbA1c (lower than 0.2%), whereas baseline lipid traits showed the opposite pattern, explaining more variance in HbA1c (higher than 6.0%) than in OGTT‐derived glucose traits (range from 3.5% to 3.9%). These findings suggest that gut microbial alterations and host lipid profiles may reflect complementary dimensions of later glycemic dysregulation.

At the species level, 26 microbial taxa were associated with later GDM risk with adjustment of maternal age, gestational age, pre‐pregnancy BMI, lifestyle factors, reproductive factors, and family history of T2DM (false discovery rate adjusted *p* value [FDR] < 0.05, Figure 2D and Table S2). These associations were highly consistent in a sensitivity analysis further adjusting for a history of polycystic ovary syndrome (PCOS). Of these, 18 species were inversely associated with GDM risk, whereas eight showed positive associations. GDM‐associated taxa were characterized by depletion of several Firmicutes species with established metabolic relevance, including *Ruminococcus bromii* [17] and *Blautia wexlerae* [18], and enrichment of species such as *Bacteroides caccae* [19]. Several GDM‐protective species were also associated with host lipid‐related traits in early pregnancy (Figure S1), supporting a potential link between gut microbial composition and metabolic regulation.

To assess the broader metabolic relevance of these microbial signatures, we compared them with findings from a large external meta‐analysis of T2DM metagenomes (*n* = 8117, with 1851 T2DM patients) [16]. Seven of the GDM‐associated species showed concordant associations with T2DM independent of age, sex, and BMI in independent populations (*p* < 0.05, Figure 2E). Among these, several species showed relatively larger effect sizes, such as *Fusicatenibacter saccharivorans, Ruminococcus bicirculans*, and *Anaerostipes hadrus*. These results suggest that part of the early‐pregnancy gut microbial signature associated with later GDM may reflect a broader diabetes‐related pattern of metabolic dysregulation.

**Figure 3:**
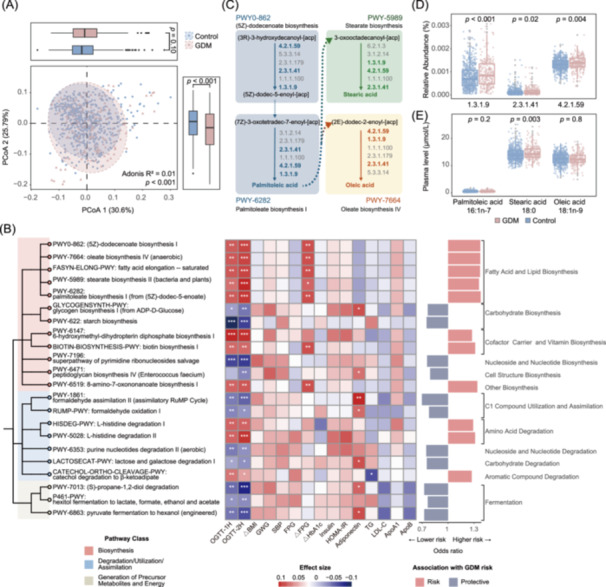
Microbial functional features linked to the risk of GDM. (A) Principal coordinate analysis of gut microbial functional composition based on Bray‐Curtis distance using MetaCyc pathway profiles. (B) Associations of microbial functional pathways with later GDM and related clinical traits. Heatmap colors and bar heights represent effect estimates from multivariable‐adjusted models. (C) Schematic overview of the fatty acid biosynthesis pathways most strongly associated with later GDM, illustrating the generation of palmitoleic acid and its conversion to stearic acid and oleate. (D) Key enzymes within the fatty acid biosynthesis pathways associated with later GDM. (E) Associations of the corresponding circulating fatty acids with later GDM. EC, Enzyme Commission; GWG, gestational weight gain; HOMA‐IR, homeostatic model assessment for insulin resistance. *FDR < 0.25; **FDR < 0.10; ***FDR < 0.05.

Consistent with the taxonomic shifts, microbial functional potential also differed between women who did and did not later develop GDM (PERMANOVA *p* = 0.0001, Figure 3A). A total of 22 microbial pathways were independently associated with GDM risk with multivariable adjustment (FDR < 0.05, Figure 3B and Table S3). The functional profile associated with later GDM risk was characterized by increased capacity for fatty acid and lipid biosynthesis, together with reduced capacity for fermentation and carbohydrate and nucleotide metabolism. Notably, pathways involved in fatty acid biosynthesis showed the strongest positive associations with GDM risk (Figure 3B), and these pathways collectively generate palmitoleic acid (16:1 *n*‐7) and further convert it to stearic acid (18:0) and oleic acid (18:1 *n*‐9) (Figure 3C), fatty acids that have been linked to insulin resistance and metabolic dysfunction in T2DM [20]. Furthermore, key enzymes within these pathways were primarily contributed by GDM‐associated species such as *Bacteroides plebeius* (Figures 3D and S2). By contrast, among the corresponding circulating fatty acids, only stearic acid was positively associated with GDM risk (odds ratio [OR] = 1.29, *p* = 0.003, Figure 3E), while palmitoleic acid and oleate were not. This discrepancy suggests that microbiome‐associated alterations in lipid metabolism may not be fully captured by the corresponding circulating fatty acids alone, prompting further investigation of the broader plasma lipidome, including more complex lipid metabolites.

### Distinct plasma lipidomic alterations were linked to gut microbial features and later GDM

**Figure 4:**
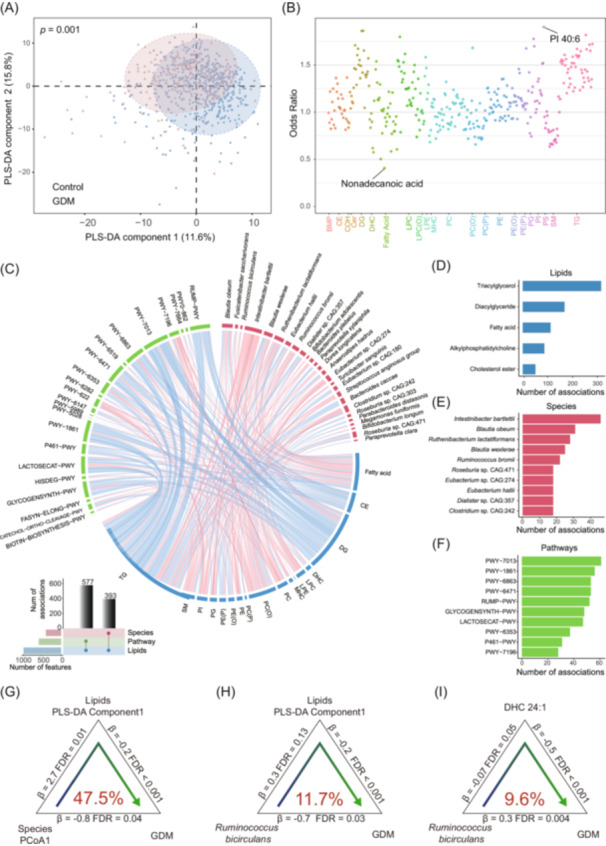
Plasma lipidomic alterations linked the early‐pregnancy gut microbiome to later GDM. (A) Partial least squares discriminant analysis of plasma lipidomic profiles in women who later developed GDM and those who remained normoglycemic. Statistical significance was assessed by permutation testing (*n *= 999). (B) Lipid metabolites associated with later GDM after multivariable adjustment (FDR < 0.05). (C) Trans‐omics network showing significant associations between GDM‐related gut microbial features and GDM‐related plasma lipid metabolites. Red lines indicate positive associations, and blue lines indicate inverse associations. (D–F) Major contributors to the trans‐omics associations among lipid metabolites (D), microbial species (E), and microbial functional pathways (F), ranked by the number of significant associations. (G–I) Mediation analyses illustrating the potential role of plasma lipidomic variation in linking gut microbial features to later GDM, including overall mediation by global lipidomic variation (G), mediation of the *Ruminococcus bicirculans*‐GDM association through overall lipidomic variation (H), and mediation through DHC 24:1 (I). BMP, bis(monoacylglycero)phosphate; CE, cholesterol ester; Cer, ceramide; COH, cholesterol; DG, diacylglyceride; DHC, dihexosylceramide; LPC, lysophosphatidylcholine; LPE, lysophosphatidylethanolamine; MHC, monohexosylceramide; PC, phosphatidylcholine; PE, phosphatidylethanolamine; PG, phosphatidylglycerol; PI, phosphatidylinositol; PLS‐DA, partial least squares discriminant analysis; PS, phosphatidylserine; SM, sphingomyelin.

To further determine whether these microbiome‐associated alterations in lipid metabolism were reflected in the broader circulating lipidome, we performed targeted plasma lipidomic profiling at baseline (see Methods). After quality control, 328 lipid metabolites were retained for analysis. Overall lipidomic profiles differed significantly between women who later developed GDM and those who remained normoglycemic, as shown by partial least squares discriminant analysis (PLS‐DA; cross‐validation with 999 permutations, *p* = 0.001, Figure 4A). Among these lipid metabolites, 165 lipids across 17 classes or subclasses were associated with GDM risk after multivariable adjustment (FDR < 0.05, Figure 4B and Table S4), indicating that widespread lipid dysregulation was already present in early pregnancy before clinical diagnosis.

The lipid alterations associated with later GDM were characterized by higher levels of multiple glycerolipid metabolites, particularly triglycerides (TG), diacylglycerides (DG) and lysophosphatidylcholines (LPC), whereas inverse associations were observed for several phosphatidylcholines (PC) and sphingomyelins (SM) (Figure 4B). Notably, the magnitude of association varied across lipid classes and according to acyl‐chain carbon number (Figure S3). Overall, these associations followed a U‐shaped pattern, with shorter‐chain lipids such as dihexosylceramide (DHC) and cholesterol ester (CE) tending to show lower ORs for GDM as carbon number increased, whereas longer‐chain lipids, particularly TG, showed the opposite trend. This pattern suggests that the relevant metabolic perturbation involved selective remodeling of specific lipid metabolites rather than total lipid burden alone.

We next examined the relationship between gut microbial features and the plasma lipidome. Global profiles of the gut microbiome and lipidome were significantly correlated by Procrustes analysis (Monte Carlo *p* = 0.03, Figure S4). Feature‐level trans‐omics analysis further identified 970 significant associations involving 26 microbial species, 22 pathways, and 126 lipid metabolites (FDR < 0.25, Figures 4C, S5, and S6). TG and DG accounted for the largest proportion of these microbiome‐lipid associations, while several GDM‐associated microbial features, including *Intestinibacter bartlettii* and fermentation‐related pathways, emerged as major contributors (Figure 4D–F). These results indicate that early‐pregnancy gut microbial variation is closely linked to host lipidomic heterogeneity, particularly in lipid classes relevant to insulin resistance and metabolic dysfunction.

To further assess whether lipid alterations might statistically link the microbiome to later GDM risk, we performed exploratory bidirectional mediation analyses within microbiome‐lipid‐GDM co‐associated groups. At the global level, plasma lipidomic variation statistically accounted for nearly half of the association between overall gut microbial composition and GDM risk (mediation proportion = 47.5%, *p‐forward* = 0.02 and *p‐inverse* = 0.35, Figure 4G). At the individual feature level, 259 microbiome‐lipid‐GDM linkages were prioritized in the forward direction (FDR*‐forward* < 0.25 and *p‐inverse* ≥ 0.05, Table S5), with DHC 24:1 emerging as the most prominent mediator. DHC 24:1 accounted for the largest number of microbial‐GDM associations (*n* = 13, Table S5), and *R. bicirculans* showed particularly consistent mediation signals through both overall lipidomic variation and this individual lipidomic feature (Figure 4H,I). These findings prioritized a candidate microbiome‐glycosphingolipid interaction axis for downstream experimental evaluation.

### Experimental evaluation of a candidate *R. bicirculans*‐glycosphingolipid axis

We further attempted to functionally evaluate, *in vitro* and *in vivo*, a candidate *R. bicirculans*‐glycosphingolipid axis prioritized by the population analyses. DHC 24:1 is a class‐level annotation that includes multiple structural isomers, such as lactosylceramide (LacCer)‐related metabolites [21]. Here, we chose LacCer 24:1 as a biologically plausible representative candidate of DHC 24:1 for downstream experimental evaluation, given its biological relevance in mammalian glycosphingolipid metabolism [22] and commercial availability.

**Figure 5:**
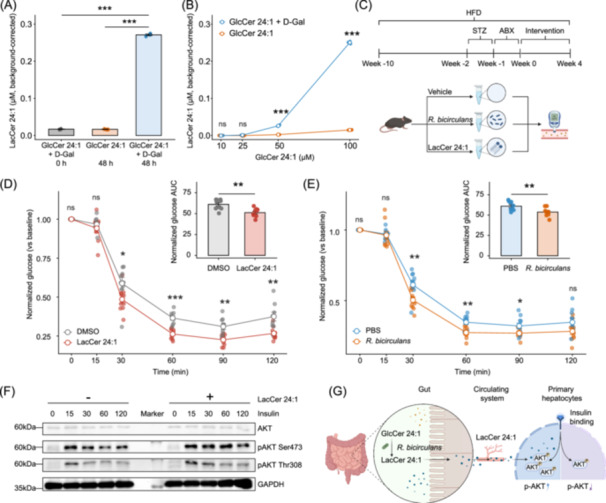
Experimental evaluation of a prioritized *Ruminococcus bicirculans*‐glycosphingolipid axis. (A) Production of LacCer 24:1 after anaerobic incubation of *R. bicirculans* under different substrate conditions. (B) Dose‐dependent production of LacCer 24:1 by *R. bicirculans* in the presence of increasing concentrations of glucosylceramide (GlcCer) 24:1, with or without d‐galactose supplementation. (C) Schematic overview of the *in vivo* intervention experiment. Mice were subjected to high‐fat diet feeding, streptozotocin (STZ) treatment, antibiotic‐mediated microbiota depletion, and subsequent intervention with vehicle, *R. bicirculans* or LacCer 24:1. (D) Insulin tolerance test (ITT) curves and corresponding area under the curve (AUC) in mice following LacCer 24:1 administration. (E) ITT curves and corresponding AUC in mice following *R. bicirculans* colonization. AUCs were calculated using the trapezoidal rule. (F) Representative Western blots showing the time course of insulin‐stimulated AKT phosphorylation in primary hepatocytes pretreated with LacCer 24:1 or vehicle. (G) Schematic model of the proposed *R. bicirculans*‐glycosphingolipid‐host interaction axis. *R. bicirculans* may contribute to LacCer‐related metabolite production, which is associated with modulation of insulin‐stimulated AKT signaling in hepatocytes. Data are presented as mean ± SEM where applicable. Statistical significance was assessed using two‐sided tests as indicated *p < 0.05; **p < 0.01; ***p < 0.001; ns, not significant. The schematic was created with BioRender.com. ABX, antibiotics; d‐Gal, d‐galactose; DMSO, dimethyl sulfoxide; GAPDH, glyceraldehyde‐3‐phosphate dehydrogenase; GlcCer, glucosylceramide; HFD, high‐fat diet; ITT, insulin tolerance test; pAKT, phosphorylated AKT; PBS, phosphate‐buffered saline; SEM, standard error of the mean; STZ, streptozotocin.

We first tested whether *R. bicirculans* could produce LacCer 24:1 under anaerobic conditions. In the presence of both glucosylceramide (GlcCer) 24:1 as substrate and d‐galactose as a co‐substrate, *R. bicirculans* generated abundant LacCer 24:1 after 48 h (Figure 5A), and the LacCer 24:1 production increased in a substrate‐dependent manner across increasing concentrations of GlcCer 24:1 (Figure 5B). These findings support the capacity of *R. bicirculans* to generate LacCer 24:1 *in vitro*.

Next, we evaluated the metabolic relevance of LacCer 24:1 in the context of diabetes. In a high‐fat diet/streptozotocin (STZ)‐induced diabetes model following antibiotic‐mediated microbiota depletion (Figure 5C), both LacCer 24:1 administration and *R. bicirculans* colonization improved insulin tolerance, as reflected by lower glucose levels over time and reduced area under the curve relative to controls in the insulin tolerance test (Figure 5D,E). Body weight and most circulating lipid parameters remained broadly unchanged during the intervention period (Figures S7 and S8), suggesting that the observed metabolic effects were not explained by major differences in overall adiposity or systemic lipid levels.

Finally, we examined whether LacCer 24:1 influenced insulin‐responsive signaling in primary hepatocytes. Cells pretreated with LacCer 24:1 and subsequently stimulated with insulin showed enhanced phosphorylation of AKT at Thr308 and Ser473 over time, whereas total AKT abundance remained unchanged (Figure 5F). These results suggest that LacCer 24:1 modulates insulin‐stimulated AKT signaling dynamics in hepatocytes.

Taken together, these experiments provide functional support for a candidate microbiome‐glycosphingolipid‐host interaction axis involving *R. bicirculans*. While not establishing a complete causal chain, these findings are consistent with the hypothesis that microbiome‐associated glycosphingolipid metabolism contributes to host metabolic regulation (Figure 5G).

### Characterizing microbial and lipidomic features in early pregnancy improved risk stratification of GDM

**Figure 6:**
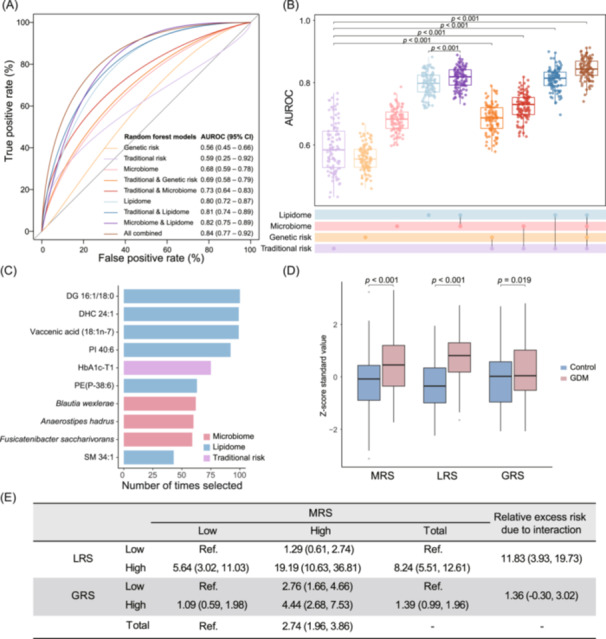
Integration of microbiome and lipidomic features improved early GDM risk stratification. (A) Receiver operating characteristic curves for random forest models predicting later GDM using baseline microbial, lipidomic, genetic, and clinical features (i.e., maternal age, BMI, physical activity levels, FPG, HbA1c, blood pressure, history of polycystic ovary syndrome, family history of T2DM, and parity), individually or in combination. (B) Predictive performance of the models, summarized by the area under the receiver operating characteristic curve. (C) Top contributors to the integrated prediction model, ranked by number of times selected across repeated cross‐validation. (D) Microbial, lipid, and genetic risk scores in women who later developed GDM and those who remained normoglycemic. (E) Odds ratios for later GDM according to joint categories of microbial, lipid, and genetic risk scores, together with the corresponding additive interaction estimates. CI, confidence interval; GRS, genetic risk score; LRS, lipid risk score; MRS, microbial risk score; PE(P), alkenylphosphatidylethanolamine; ROC, receiver operating characteristic.

To assess the potential translational relevance of the identified multi‐omics features, we next evaluated whether gut microbial, lipidomic, and genetic markers measured in early pregnancy could improve risk stratification for later GDM beyond established clinical factors. Random forest models based on individual omics layers showed moderate discriminatory performance, with the lipidome model achieving the highest performance among single‐layer models (area under the receiver operating characteristic curve [AUROC] 0.80, 95% CI 0.72 to 0.87), outperforming both the microbiome model (0.68, 0.59 to 0.78) and the model based on traditional clinical risk factors alone (0.59, 0.25 to 0.92) (both *p*‐difference < 0.05, Figure 6A,B). Adding microbiome data to the lipidome model further improved discrimination, yielding an AUROC of 0.82 (95% CI 0.75 to 0.89). The fully combined model incorporating all omics layers together with clinical factors achieved the best overall discrimination (AUROC 0.84, 95% CI 0.77 to 0.92, Figure 6B). In the integrated model, several microbial species and lipid metabolites contributed prominently to predictive performance, including *B. wexlerae*, *A. hadrus*, and *F. saccharivorans* (Figure 6C). Notably, these microbial taxa overlapped with the features implicated in the microbiome‐lipid analyses above, supporting the relevance of the identified metabolic axis to early risk stratification.

To further summarize the joint effects of the multi‐omics features, we constructed composite microbial, lipid, and genetic risk scores based on selected microbial species, lipid metabolites, and reported GDM‐associated variants (see Methods). Women who later developed GDM had higher microbial and lipid risk scores than those who remained normoglycemic (*p* < 0.05, Figure 6D). Higher microbial and lipid risk scores were each associated with increased GDM risk, and women with concurrently elevated microbial and lipid scores had an OR of 19.19 for GDM (95% CI 10.63 to 36.81) compared with those with low scores for both domains (Figure 6E). A significant additive interaction was observed between the microbial and lipid risk scores (relative excess risk due to interaction = 11.83, *p‐interaction* = 0.003), indicating that the combined effect exceeded the sum of their individual contributions. Together, these findings suggest that integrating microbiome and lipidomic information captured in early pregnancy may help refine GDM risk stratification, while also supporting the biological relevance of the microbiome‐lipid signatures identified in the primary analyses.

## DISCUSSION

In this prospective study of 784 pregnant women, we identified taxonomic and functional features of the early‐pregnancy gut microbiome associated with later GDM and characterized their relationships with the circulating lipidome. Integrative analyses highlighted a microbiome‐lipid axis linked to metabolic dysregulation in pregnancy, with glycosphingolipid‐related metabolites emerging as potential mediators of the association between gut microbial variation and later GDM risk. We further prioritized an *R. bicirculans*‐glycosphingolipid axis and provided experimental evidence consistent with its potential relevance to host glucose regulation and insulin‐responsive signaling. In addition, integration of microbial and lipidomic features improved early risk stratification beyond established clinical factors. Together, these findings suggest that gut microbiome‐associated lipid metabolism may contribute to early metabolic maladaptation in pregnancy.

Previous studies have linked GDM to alterations in gut microbial composition, microbial metabolites, circulating metabolomic profiles, inflammatory pathways, and pregnancy hormone metabolism [9, 10, 23, 24, 25], whereas our study further integrates prospective metagenomic and lipidomic profiling with experimental follow‐up of a prioritized microbiome‐glycosphingolipid axis. We previously reported that early‐pregnancy lipidomic profiles are associated with subsequent GDM risk [8], and that the gut microbiome dynamically relates to host glucose metabolism across pregnancy [26]. Here, by integrating metagenomic and lipidomic data, we show that the early‐pregnancy gut microbiome was linked not only to later GDM risk but also to coordinated alterations in the circulating lipidome. Microbial features and lipid metabolites were strongly correlated, and part of the microbiome‐GDM association was statistically mediated through lipidomic variation, supporting the concept that host lipid metabolism may represent an important interface through which gut microbial function is translated into metabolic phenotypes during pregnancy [11]. Consistent with this interpretation, women who later developed GDM showed enrichment of microbial pathways related to fatty acid and lipid biosynthesis and relative depletion of fermentation‐ and carbohydrate‐related pathways, suggesting a shift in microbial metabolic capacity towards lipid‐producing functions [12]. Fatty acid biosynthesis pathways collectively generate palmitoleic acid and related downstream fatty acids, including stearic acid and oleate, which have been implicated in insulin resistance and metabolic dysfunction [27]. However, among the corresponding circulating fatty acids, only stearic acid was associated with later GDM, suggesting that microbiome‐associated functional alterations may be reflected more clearly in complex lipid metabolites than in the corresponding circulating fatty acids alone. This interpretation is biologically plausible because fatty acids, diacylglycerols, ceramide‐related metabolites, and broader lipidome remodeling have been implicated in hepatic and systemic insulin resistance [28, 29, 30, 31].

Among the prioritized mediators, DHC 24:1 emerged as a representative glycosphingolipid‐related metabolite linking gut microbial variation to later GDM risk. Because the population lipidomics signal was defined at the class level, we selected LacCer 24:1 as a biologically plausible representative candidate for downstream experiments [21], rather than as a confirmed molecular identity of the human plasma DHC 24:1 signal. This choice is supported by the central role of LacCer in mammalian glycosphingolipid metabolism and by growing evidence linking sphingolipid pathways to insulin resistance and hepatic metabolic dysfunction [22, 32, 33]. In addition, bacterial sphingolipids can enter host metabolic pathways and influence host sphingolipid homeostasis, supporting the plausibility of host–microbiome interactions through sphingolipid‐related metabolism [15, 34]. Within this framework, our *in vitro* and *in vivo* experiments provide functional evidence consistent with an *R. bicirculans*‐glycosphingolipid‐host interaction axis. Specifically, *R. bicirculans* promoted LacCer‐related metabolite production under anaerobic conditions, while bacterial colonization and metabolite administration improved glucose disposal *in vivo*, and LacCer 24:1 treatment modulated insulin‐stimulated AKT signaling dynamics in hepatocytes. Although these experiments do not establish a complete causal chain, they support the biological plausibility that microbiome‐associated glycosphingolipid metabolism contributes to host metabolic regulation. The overlap between our GDM‐associated microbial signatures and T2DM‐associated taxa in external datasets further suggests that this prioritized axis may reflect a broader mechanism of diabetes‐related metabolic dysregulation rather than a process restricted to pregnancy alone [16].

Our findings also have scientific and potential translational relevance. Scientifically, this study extends previous microbiome and lipidomic observations in GDM by integrating prospective population‐level multi‐omics analyses with experimental follow‐up of a prioritized microbiome‐glycosphingolipid axis. This design allowed us to move from association‐based feature discovery toward a biologically interpretable framework in which gut microbial variation may be linked to host metabolic dysregulation through specific lipid‐related molecular intermediates. Practically, although established clinical factors remain central to early GDM assessment [5, 6], incorporation of microbiome and lipidomic features improved risk stratification beyond conventional markers. We view this predictive signal as complementary to, rather than separate from, the underlying biology, because several microbial contributors to model performance overlapped with taxa implicated in the microbiome‐lipid analyses. In this context, multi‐omics integration may be useful not only for identifying women at increased metabolic risk in early pregnancy, but also for prioritizing microbiome‐ and lipid‐related pathways for future validation, mechanistic investigation, and prevention‐oriented studies. Nevertheless, clinical implementation would require independent validation in diverse pregnancy cohorts, standardized and scalable assays, cost‐effectiveness evaluation, and assessment of feasibility in routine prenatal care.

This study has several strengths, including prospective sampling in early pregnancy, integration of shotgun metagenomics and targeted lipidomics, and experimental follow‐up of a prioritized microbiome‐glycosphingolipid axis. Several limitations should also be acknowledged. The observational design limits causal inference in the human cohort; the study population was recruited from western China and consisted predominantly of Han Chinese pregnant women, which may limit the generalizability of our findings to other geographic regions and ethnic groups; microbiome and lipidome measurements were obtained at a single time point; residual confounding by diet cannot be fully excluded, as dietary information was collected using a brief questionnaire on selected food groups rather than a comprehensive food‐frequency questionnaire or repeated dietary records. Given the close relationships among diet, gut microbiome, and host meta‐metabolomic profiles, future studies with more comprehensive dietary assessment are needed to better disentangle dietary, microbial, and lipidomic contributions to GDM risk [35]. The small proportion of variance explained by the measured dietary and lifestyle variables may reflect both the limited resolution of the questionnaire‐based assessment and the multifactorial determinants of microbiome and lipidome variation. In addition, independent external validation of the full multi‐omics findings, particularly lipidomic associations and risk stratification models, was not available. The random forest models were evaluated using repeated internal cross‐validation; however, high‐dimensional omics features may still be susceptible to overfitting and multicollinearity. Therefore, the reported AUROC values should be interpreted as internally cross‐validated estimates of risk stratification performance rather than evidence of clinical generalizability. Although the experimental data support biological plausibility, they do not fully resolve the structural identity of the human lipid signal or define the complete molecular steps linking microbial metabolism to host insulin‐responsive signaling. In particular, the DHC 24:1 feature identified in the population lipidomics analysis was annotated at the class level and may comprise multiple structural isomers. Although LacCer 24:1 was selected as a biologically plausible representative candidate for experimental follow‐up, our data do not establish that the human plasma DHC 24:1 signal primarily reflects LacCer 24:1. Future studies using higher‐resolution lipid structural characterization and targeted LC‐MS/MS validation in clinical samples are needed to determine the exact isomeric composition of this feature and to evaluate whether different DHC 24:1 isomers exert distinct metabolic effects. In addition, the *in vivo* experiment was conducted in male HFD/STZ‐induced mice and therefore does not recapitulate the gestational endocrine, placental, and immune environment of human GDM. Thus, the animal experiment should be interpreted as a functional plausibility assessment under insulin resistance‐related metabolic dysfunction, rather than as a pregnancy‐specific model of GDM.

## CONCLUSION

In conclusion, our study supports a role of the gut microbiome in metabolic dysregulation during pregnancy, particularly through its links with host lipid metabolism. By integrating metagenomic, lipidomic, and experimental data, we identified a microbiome‐lipid axis associated with later GDM and prioritized a glycosphingolipid‐related pathway that may connect microbial function to host insulin‐responsive signaling. These findings extend current understanding of the microbiome‐host metabolic interface in pregnancy and highlight potential targets for early risk stratification and mechanistic investigation.

## METHODS

### Study design and population

This study was conducted within the Tongji‐Huaxi‐Shuangliu Birth Cohort (THSBC, previously the Tongji‐Shuangliu Birth Cohort), an ongoing prospective cohort in Shuangliu District, Chengdu, China [8]. Pregnant women aged 18–40 years who attended their first prenatal examination before 15 weeks of gestation were recruited. At baseline (T1), all enrolled participants were required to provide blood and fecal samples and complete a lifestyle questionnaire. GDM was diagnosed during T2 (24–28 weeks of gestation) using a 75‐g oral glucose tolerance test (OGTT) according to the criteria established by the International Association of the Diabetes and Pregnancy Study Groups (i.e., fasting 5.1 mmol/L, 1‐h 10.0 mmol/L, and 2‐h 8.5 mmol/L) [36].

As previously described [8], a nested case‐control sample was constructed within the cohort, including 336 women who developed GDM and 672 women who remained normoglycemic during follow‐up, with each GDM case individually matched to two controls by maternal age (±3 years) and gestational age at recruitment (±4 weeks). For the current analysis, women were further excluded if they had a prior diagnosis of T2DM or failed to provide fecal samples in the first trimester. The final analytic sample, therefore, comprised 222 women who developed GDM and 562 women who remained normoglycemic during follow‐up (Figure 1). Baseline characteristics and clinical measurements of included and excluded GDM cases were summarized in Table S6. In the present study, baseline omics profiles were measured before clinical diagnosis of GDM, and GDM was analyzed as a longitudinal outcome.

### Biochemical measurements and clinical data collection

Plasma glucose was measured by the Glucose Assay Kit (Maccura Biotechnology) using the GOD‐PAP (glucose oxidase‐phenol and 4‐aminophenazone) method. Glycated hemoglobin (HbA1c) was measured by DCA Vantage Analyzer (Siemens Healthcare Diagnostics). Fasting serum insulin was measured by Meso Scale Discovery (MSD) U‐PLEX Metabolic Assays. Homeostatic model assessment for insulin resistance (HOMA‐IR) was calculated using the following equations: [fasting glucose (mmol/L) × fasting insulin (mU/L)]/22.5 [37]. High‐density lipoprotein cholesterol (HDL‐C), low‐density lipoprotein cholesterol (LDL‐C), triglycerides, and total cholesterol were measured using a commercial BS‐200 automatic biochemistry analyzer kit (Mindray). The measurements of anthropometrics and covariates (i.e., body mass index [BMI], blood pressure, physical activity, reproductive factors, and disease history) have been described previously [38]. Gestational weight gain (GWG) was calculated as the difference between the weight in T1, T2, and pre‐pregnancy weight.

### Taxonomic and functional profiling of fecal samples

Fecal samples were collected using sterile containers with ice boxes at the hospital or home and stored at −40°C within 2 h after collection and then were transported with dry ice to the central laboratory and stored at −80°C until processing.

The detailed process of DNA extraction and microbial profiling has been described previously [26, 39]. In brief, fecal DNA was extracted using the TIANamp Stool DNA kit (TIANGEN) according to the experimental protocols [40]. Illumina sequencing libraries (paired‐end, insert size: 350 bp) were prepared using the Tn5 DNA Library Prep Kit for Illumina (APExBIO) according to the manufacturer's protocols. All libraries were sequenced on the Illumina NovaSeq 6000 platform (read length: 150 bp). The quality control process of whole‐genome shotgun sequencing data was performed by KneadData (version 0.7.2), Trimmomatic (version 0.33) [41], and Bowtie 2 (version 2.3.4.3) [42]. Human reads and rRNA reads were filtered by mapping the reads to the human reference genome (GRCh37) and the SILVA 128 database. The trimmed nonhuman reads shorter than 75 bp were also removed. The taxonomic profiles were determined by MetaPhlAn (version 3.0.3) [43], and the microbial functional profiles, including MetaCyc pathways and Enzyme Commission gene families, were determined by HUMAnN (version 3.0.0) [44]. Microbial species with a relative abundance <0.01% in over 90% samples and functional pathways with a relative abundance <0.001% in over 90% samples were removed from subsequent analysis.

In the current study, all the 784 fecal samples were successfully sequenced. After quality control, an average of 58.7 million (ranging from 16.3 to 187.8 million) high‐quality reads were obtained for each sample. Eventually, a total of 163 microbial species and 327 pathways were included in the following analyses (Tables S2 and S3).

### Lipidome and fatty acid profiling of blood samples

Details of lipidomic analysis were described previously [8]. Briefly, fasting blood samples were collected in T1 and stored at −40°C in the hospital for less than 6 months before being transported to the laboratory with −80°C preservation until further analysis. The lipid extraction method was adapted for single‐phase plasma lipid extraction based on contemporary MS‐based lipidomics workflows [45, 46]. Targeted lipidomic instrumental analysis was performed on an ultra‐HPLC‐tandem MS. In the current study, a total of 738 plasma samples were collected, 366 lipid molecular species from 24 lipid classes/subclasses were profiled, and analytes with a detection rate <80% or coefficient of variation (CV) > 20% (either in intra‐ or in inter‐assay CVs) were excluded (n = 38). Eventually, 328 lipid metabolites from 21 lipid classes/subclasses were included for further analysis (Table S4). Analytes with undetectable values (3 lipids had 5%–20% and 38 lipids had <5% undetectable values) were estimated using half of the lowest detection value.

Details of fatty acid profiling were also described previously [38]. In brief, plasma phospholipid fatty acids were measured by gas chromatography‐mass spectrometry using fasting blood samples collected at baseline. A total of 31 individual fatty acids were quantified, including even‐chain saturated fatty acids (SFAs), odd‐chain SFAs, very‐long‐chain SFAs, long‐chain monounsaturated fatty acids (MUFAs), omega‐3 (n‐3) polyunsaturated fatty acids (PUFAs), and omega‐6 (*n*‐6) PUFAs. All plasma fatty acids were expressed as the percentage of the concentration of total fatty acids. All assays were conducted without the knowledge of the case‐control status, and samples were assayed in the same analytic run in a random order.

### Genotyping

The detailed method for genotyping has been described previously [47]. Briefly, genomic DNA was extracted from peripheral blood by using a commercial DNA extraction kit (TIANGEN), and the genome‐wide genotyping was conducted using the Illumina HumanOmni ZhongHua‐8 Bead Chips. Samples with a call rate > 95% were retained. A total of 689 participants provided genomic data in the current analysis.

### Replication population

To explore whether GDM and T2DM shared microbial features, we further compared our results with a recent meta‐analysis including 8117 shotgun metagenomes from 10 cohorts of participants with T2D and normoglycemic status in the United States, Europe, Israel, and China [16].

### Statistical analysis

Baseline characteristics of the study population were presented as mean ± standard deviation (SD) for continuous variables and as *n* (%) for categorical variables. The α‐diversity of the gut microbiome was represented by Shannon and Gini‐Simpson indexes calculated using a species‐level relative abundance matrix for each sample. The β diversity of gut microbiome between samples was calculated using species‐level Bray‐Curtis distance and was visualized using principal coordinate analysis (PCoA). The differences in microbial composition between different groups or trimesters were calculated using permutational multivariate analysis of variance (PERMANOVA) using 9,999 permutations via the R package “vegan” (version 2.6‐2). The explained variance was calculated using multivariable‐adjusted linear regression. Models were adjusted for relevant baseline covariates, including maternal age, gestational age, education, parity, family history of diabetes, pre‐pregnancy BMI, cigarette smoking, alcohol drinking, physical activity, and antibiotic use, as appropriate.

The differences in lipidome profile between different groups were visualized by partial least squares‐discriminant analysis (PLS‐DA) using the first two components with R package “mixOmics” (version 6.18.1), and the statistical significance was assessed by 999 permutation tests via the function *MVA.test* in R package “RVAideMemoire” (version 0.9‐81‐2). The association between gut microbial composition and lipid profile was estimated using Procrustes analysis via the function *Procrustes* in the R package “vegan.”

The associations between microbial features (the relative abundances of microbial species and functional pathways), metabolic features (the plasma concentrations of lipidome and fatty acids), and GDM status and related metrics were estimated using MaAsLin2 (version 1.16.0) with linear models [48]. The continuous clinical measurements were scaled to *Z*‐scores. Before association analyses, centered log‐ratio transformation (CLR) and *Z*‐scores were applied to standardize the relative abundance of gut microbial species and functional pathways, and inverse normal transformation (INT) was applied to metabolic features. All models were adjusted for maternal age, gestational age, education, parity, family history of diabetes, pre‐pregnancy BMI, cigarette smoking, alcohol drinking, physical activity, and use of antibiotics. All *p*‐values for multiple hypothesis testing were adjusted for multiple comparisons using the Benjamini‐Hochberg method. For differential analysis, a false discovery rate (FDR) of less than 0.05 was considered statistically significant. For trans‐omics analysis, an FDR of less than 0.25 was deemed statistically significant.

To explore the potential role of lipids in the association between the gut microbiome and GDM risk, we performed an exploratory bidirectional mediation analysis. First, based on the hypothesis that gut microbial variation may influence host metabolic dysregulation through lipidomic changes, we evaluated whether lipidomic features mediated associations between gut microbiome features and GDM‐related phenotypes (forward mediation: microbiome‐lipidome‐GDM). *p* values for forward mediation effects were adjusted using the Benjamini‐Hochberg method, and mediation effects with FDR < 0.25 were considered significant. We then assessed the reverse directionality by testing whether gut microbiome features mediated associations between lipidomic features and GDM‐related phenotypes (reverse mediation: lipidome‐microbiome‐GDM), with nominal *p* < 0.05 considered statistically significant. The reverse mediation analysis was used as a secondary directionality check among forward‐prioritized mediation patterns, rather than as an independent feature‐discovery pipeline. To prioritize biologically interpretable mediation pathways for downstream mechanistic investigation, we focused on forward mediation patterns showing significant forward mediation but no nominally significant evidence of reverse mediation.

To evaluate the predictive value of early‐pregnancy omics features for subsequent GDM, we constructed random forest models using data collected at T1 to predict GDM diagnosed at T2. Models were first built using traditional clinical risk factors at T1 (maternal age, BMI, physical activity levels, FPG, HbA1c, blood pressure, history of polycystic ovary syndrome [PCOS], family history of T2DM, and parity) [49], and were subsequently extended by incorporating gut microbial features, circulating lipid features, and genetic risk variants (four previously reported GDM‐associated SNPs in Chinese populations; Table S7). All feature sets were defined prior to model training, and no outcome information from the test folds was used during model fitting or parameter tuning. Model performance was evaluated using repeated fivefold cross‐validation (100 repeats), in which model training and parameter tuning were performed within the training folds and the held‐out folds were used only for performance evaluation. Parameter tuning was implemented via the R package “caret” (version 6.0‐94). The AUROC was used to quantify model discrimination. Differences in AUROC between models were summarized across cross‐validation resamples and used to compare relative model performance. This procedure should be interpreted as internal cross‐validation rather than independent external validation.

To uncover synergistic interactions between fecal microbiota, plasma lipidome, and genetic predisposition, their additive interaction effects were estimated by adding an interaction term into the regression model, which consisted of microbial risk score (MRS) and lipid risk score (LRS)/genetic risk score (GRS) (categorical: lower or higher classified by median value). The logistic regression models and covariates used in the interaction analysis were the same as those used in the aforementioned analysis. A relative excess risk due to interaction larger than 0 with a *p* < 0.05 was regarded as a statistically significant additive interaction. The MRS was calculated based on the relative abundance of GDM‐related species selected through the least absolute shrinkage and selection operator (LASSO) penalized regression with 1000 repetitions of 10‐fold cross‐validation [50]. Only species with non‐zero coefficients in more than 980 repetitions were selected for MRS calculation. The LASSO‐selected species were categorized into two groups, $M_{P}$ and $M_{N}$, where $M_{P}$ was the group of species positively associated with GDM and vice versa for $M_{N}$. The MRS of sample *i* is defined as

MRS=log10RMP,i|MP|∑j∈MPxj,iRMN,i|MN|∑j∈MNxj,i,

where $R_{M_{P,i}}$ denotes the richness of $M_{P}$ (i.e., the number of present species of $M_{P}$) in sample *i*, $\left|M_{P}\right|$ is the size of group $M_{P}$ (i.e., the overall number of species in $M_{P}$), $x_{j,i}$ denotes the relative abundance of species *j* in sample *i* and the same for $R_{M_{N,i}}$ and $\left|M_{N}\right|$. The calculation of MRS considered both the richness and the relative abundance of LASSO‐selected species to quantify the balance between $M_{P}$ and $M_{N}$. Due to the difference between the set sizes of $M_{P}$ and $M_{N}$, the proportion of the present species of these two groups was calculated for each sample $\left(\frac{R_{M_{P}}}{\left|M_{P}\right|}\;\mathrm{and}\;\frac{R_{M_{N}}}{\left|M_{N}\right|}\right)$ instead of the richness $R_{M_{P}}$ and $R_{M_{N}}$. As such, a higher MRS or MRS > 0 indicates that the species profile is associated with a higher risk for GDM. Methods for the development of LRS have been described previously [8]. In brief, the candidate lipids were selected from multivariable‐adjusted logistic regression models. Subsequently, lipid biomarkers were selected through LASSO regression, with the LASSO penalty parameters being adjusted for covariates. The LRS was then formulated based on the weighted sum of the LASSO‐selected lipid concentrations to evaluate the composite effect. The GRS was created based on SNPs that were summarized from a recent genome‐wide association study (GWAS) among 30,699 Chinese women [51]. Specifically, weighted GRS was created by summing up risk alleles of the four GDM‐related SNPs multiplied by the corresponding weight estimated on the beta coefficient of each SNP with risk of GDM.

All data analyses were conducted in R (version 4.3.2). Detailed model specifications for the main analyses, including input features, outcome variables, data transformations, covariates, multiple‐testing correction, software packages, and key parameters, were summarized in Table S8.

### Anaerobic culture assay for LacCer production

*Ruminococcus bicirculans* (currently classified as *Hominimerdicola aceti*) was purchased from DSMZ (DSM 102216). The identity of the strain was confirmed by 16S rRNA gene sequencing. The strain was cultured under anaerobic conditions to evaluate its capacity to generate LacCer 24:1 from glycosphingolipid‐related substrates. Briefly, bacteria were inoculated from glycerol stocks into anaerobic culture medium and incubated at 37°C until reaching the stationary phase. Fresh bacterial cultures were then supplemented with GlcCer 24:1 (C24:1 Glucosyl(β) Ceramide [d18:1/24:1(15Z)]; Avanti, USA, Cat# 860549 P), with or without d‐galactose (KKL Med, Cat# KM15328), and incubated anaerobically for the indicated durations.

To assess substrate dependence, GlcCer 24:1 was added at different concentrations (10, 25, 50, and 100 μM) in the presence or absence of d‐galactose, and cultures were incubated for 48 h. To assess condition‐specific production, cultures were also prepared with GlcCer + d‐galactose (0 h), GlcCer alone (48 h), and GlcCer + d‐galactose (48 h). After incubation, culture supernatants were collected for targeted quantification of LacCer 24:1. LacCer levels were corrected against baseline values before downstream analysis.

### Quantification of LacCer 24:1 in bacterial culture supernatants

The concentration of LacCer 24:1 in bacterial culture supernatants was quantified using a targeted liquid chromatography‐tandem mass spectrometry (LC‐MS/MS) approach. Briefly, samples were thawed slowly at 4°C, and an aliquot of the bacterial culture was centrifuged to obtain the supernatant. The supernatant was mixed with methanol containing C24:1 Lactosyl(β) Ceramide‐d7 (d18:1/24:1(15Z)) as a stable isotope‐labeled internal standard (Avanti, Cat# 860739 P), vortexed thoroughly, and centrifuged at 12,000 rpm for 10 min at 4°C. The resulting supernatant was subjected to LC‐MS/MS analysis.

Chromatographic separation was performed on an AB SCIEX Exion LC system equipped with a C18 column (Waters XBridge BEH C18, 100 × 2.1 mm, 2.5 μm). The column temperature was maintained at 50°C, the flow rate was 0.4 ml/min, and the injection volume was 5 μl. The mobile phases consisted of acetonitrile/water (2:8, v/v) for phase A and acetonitrile/isopropanol (2:8, v/v) for phase B. The gradient program was as follows: 0–4.0 min, 40% to 100% B; 4.0–5.5 min, 100% B; 5.5–5.55 min, 100% to 40% B; and 5.55–6.5 min, 40% B. Samples were kept in the autosampler at 4°C throughout the analysis.

Mass spectrometric detection was performed on a SCIEX Triple Quad 6500+ mass spectrometer operating in positive electrospray ionization mode with multiple reaction monitoring (MRM). The ion source parameters were set as follows: ion source gas 1, 55 psi; ion source gas 2, 55 psi; source temperature, 300°C; curtain gas, 30 psi; collision gas, 6 psi; and ion spray voltage, 5500 V. Raw MRM data were processed using MultiQuant version 3.0.3 software. Quantification of LacCer 24:1 was based on the peak area ratio of analyte to internal standard, using an authentic unlabeled C24:1 Lactosyl(β) Ceramide (d18:1/24:1) standard (Avanti, Cat# 860597 P) for calibration.

### Mouse model and interventions

Male C57BL/6J specific‐pathogen‐free mice (4–5 weeks old) were purchased from GemPharmatech Co., Ltd. (Nanjing, China) and acclimatized for 1 week before experiments. Mice were fed a high‐fat diet (HFD; XTHF60‐1, Jiangsu Xietong Pharmaceutical Bioengineering Co., Ltd.; 60% kcal from fat) for 8 weeks, followed by intraperitoneal injection of STZ (MedChemExpress, Cat# HY‐13753; 40 mg/kg) for 5 consecutive days to induce metabolic dysfunction. After hyperglycemia was confirmed, mice were treated with a four‐antibiotic cocktail once daily for 7 days to deplete the endogenous microbiota.

Mice were then randomized on the basis of blood glucose and body weight into four groups (*n* = 10 per group): LacCer 24:1 treatment, vehicle control, *R. bicirculans* colonization and PBS control. *R. bicirculans* was cultured anaerobically to stationary phase, washed, and resuspended in sterile PBS, and administered by oral gavage (~10^8^ CFU in 200 μl) once every 3 days for 4 weeks. PBS was used as the corresponding gavage control. LacCer 24:1 was dissolved in 10% DMSO and administered by intraperitoneal injection (10 μg per mouse in 200 μl) once every 3 days for 4 weeks. Vehicle‐treated mice received 10% DMSO alone.

### Phenotypic measurements and tissue collection

Body weight and random blood glucose were monitored longitudinally. Fecal samples were collected at baseline and weekly during intervention. ITT was performed after a 6‐h fast using intraperitoneal insulin injection (0.75 IU/kg), and OGTT was performed after a 6‐h fast using oral glucose gavage (2 g/kg). Blood glucose was measured at 0, 15, 30, 60, 90, and 120 min for both tests. AUCs for ITT and OGTT were calculated using the trapezoidal rule. At the end of the experiment, mice were euthanized by carbon dioxide inhalation, and blood, cecal contents, liver, pancreas and adipose tissues were collected for downstream analyses.

### Lipid profile assays

Blood samples were collected after euthanasia and centrifuged at 3000 rpm for 20 min at 4°C to obtain serum. The supernatant was transferred to a new tube and centrifuged again at 3000 rpm for 5 min at 4°C. The final supernatant was collected as serum samples for analysis. Serum levels of triglycerides (TG), total cholesterol (TC), HDL‐C, and LDL‐C were measured using commercial kits according to the manufacturers’ instructions. The assay principle is based on the colorimetric detection of quinone compounds, the intensity of which is proportional to the lipid concentration. Absorbance was recorded using a spectrophotometer, and lipid concentrations were calculated based on standard curves. All kits were purchased from Nanjing Jiancheng (China): TG (Cat# A110‐1‐1), TC (Cat# A111‐1‐1), LDL‐C (Cat# A113‐1‐1), and HDL‐C (Cat# A112‐1‐1).

### Isolation and culture of primary hepatocytes

Primary hepatocytes were isolated from male C57BL/6J mice (8 weeks old). Mice were anesthetized with sodium pentobarbital (50 μl/10 g body weight). The portal vein was cannulated with a 24 G indwelling needle, and the liver was perfused in situ with KRG buffer (Krebs‐Ringer with glucose) containing 0.1 mM EGTA at a flow rate of 60 rpm using a peristaltic pump. After the liver was completely blanched, the perfusion was switched to KRG buffer containing 0.5 mg/ml collagenase D (Roche, Cat# 11088858001) and 2 mM CaCl_2_ at 50 rpm. The digested liver was transferred to a dish, minced, and gently pipetted to release hepatocytes. The cell suspension was filtered through a 70 μm cell strainer and centrifuged at 50*g* for 5 min. The pellet was resuspended in isolation medium and further purified by two additional centrifugations at 60*g* for 3 min and 700 rpm for 2 min. Isolated hepatocytes were resuspended in DMEM (1 g/L glucose) supplemented with 10% FBS and 1% antibiotics, seeded onto collagen‐coated plates (30 μg/ml rat tail collagen, Sigma‐Aldrich, Cat# C3867‐1VL), and cultured at 37°C in 5% CO_2_. Cells were allowed to attach for 4–6 h before further treatment.

### Cell treatment

Primary hepatocytes were treated with 10 μM LacCer 24:1 (Avanti, Cat# 860597 P) or vehicle overnight after plating. Primary hepatocytes were then stimulated with 100 nM insulin (Beyotime, Cat# P3376‐100IU) or vehicle for the indicated times. Primary hepatocytes were collected at 0, 15, 30, 60, and 120 min after stimulation. After stimulation, the medium was removed, and cells were lysed on ice for 30 min in RIPA buffer (strong, Beyotime, Cat# P0013B) supplemented with protease inhibitor cocktail (Beyotime, Cat# P1005) and phosphatase inhibitor cocktail C (Beyotime, Cat# P1091). Lysates were centrifuged at 12,000*g* for 15 min at 4°C, and the supernatants were collected. Protein concentrations were determined using the BCA assay (Pierce™ BCA Protein Assay Kit; Thermo Fisher, Cat# 23225).

### Western blotting

Equal amounts of protein (20 μg) were separated by SDS‐PAGE and transferred to PVDF membranes (Millipore, Cat# IPVH00010). Membranes were blocked with 5% non‐fat milk (Sangon Biotech, Cat# A600669‐0250) for 2 h at room temperature and then incubated with primary antibodies overnight at 4°C. Antibody information is provided in Table S9. Membranes were washed three times with TBST and incubated with HRP‐conjugated secondary antibodies for 1 h at room temperature. After three washes with TBST, signals were detected using ECL substrate (SuperSignal™ West Pico PLUS, Thermo Fisher, Cat# 34577) and visualized with a chemiluminescence imaging system. Band intensities were quantified using ImageJ software. Target protein expression levels were normalized to GAPDH and expressed relative to control.

## AUTHOR CONTRIBUTIONS

**Yan Zheng:** Conceptualization; supervision; writing—review and editing. **Xiong‐Fei Pan:** Conceptualization; investigation; supervision; writing—review and editing. **Shangyu Hong:** Conceptualization; validation; writing—review and editing. **Da Chen:** Conceptualization; investigation; writing—review and editing. **Zhonghan Sun:** Writing—original draft; formal analysis; data curation; validation. **Chang He:** Writing—original draft; formal analysis; data curation. **Xiaonan Ma:** Writing—original draft; validation. **Ping Wu:** Writing—original draft; investigation. **Tianlei Wang:** Investigation. **Jiaying Yuan:** Investigation. **Yanni Pu:** Writing—review and editing. **Xiaofeng Zhou:** Writing—review and editing. **Zhendong Mei:** Writing—review and editing. **Huiling Song:** Data curation. **Yetong Wang:** Data curation. **Haiyan Yue:** Data curation. **Yuanqing Fu:** Investigation. **Jusheng Zheng:** Investigation. **An Pan:** Writing—review and editing. All authors have read the final manuscript and approved it for publication.

## CONFLICT OF INTEREST STATEMENT

The authors declare no conflicts of interest.

## ETHICS STATEMENT

The THSBC study was approved by the Ethics Committee of Tongji Medical College, Huazhong University of Science and Technology (No. [2017](S225)−1), and written informed consent was obtained from all participants at recruitment. All animal experiments were approved by the Laboratory Animal Welfare and Ethics Committee of the Experimental Animal Center, Fudan University (No. YSAPL20260336).

## Supporting information


**Figure S1:** The association between microbial species and clinical measurements related to glucose and lipid metabolism.
**Figure S2:** The proportions of enzymes in microbial functional potentials of fatty acids metabolism encoded by specific species.
**Figure S3:** The associations between lipids with different numbers of acyl chain carbons and GDM risk.
**Figure S4:** Procrustes analysis of gut microbiome composition and plasma lipidome profile.
**Figure S5:** The trans‐omics associations between GDM‐related species and lipid metabolites.
**Figure S6:** The trans‐omics associations between GDM‐related microbial metabolic potentials and lipid metabolites.
**Figure S7:** Circulating lipid parameters following *in vivo* intervention.
**Figure S8:** Body weight trajectories during the *in vivo* intervention experiment.

**Table S1:** Basic characteristics and clinical measurements among cases with GDM and controls.
**Table S2:** The relative abundance and prevalence of microbial species included in the current analysis.
**Table S3:** The microbial pathways included in the current analysis.
**Table S4:** Concentrations of lipid metabolites (nmol/L) between the GDM and control groups.
**Table S5:** The mediation effect of lipids between microbial features and GDM risk.
**Table S6:** Basic characteristics of included and excluded GDM patients.
**Table S7:** The SNPs included in the genetic risk score of GDM.
**Table S8:** Summary of statistical models used in the main analyses.
**Table S9:** Antibodies used for Western blotting.

## Data Availability

All metagenomics and lipidomics data generated in this study have been deposited in NODE under the project accession number OEP00003504 (https://www.biosino.org/node/project/detail/OEP00003504) and are available upon approved access. Clinical metadata were not publicly available due to legal and informed consent restrictions. Reasonable requests to access the datasets should be directed to the corresponding authors, Xiong‐Fei Pan and Yan Zheng. The data and scripts used are available on GitHub (https://github.com/ChangHe005/SL-GDM). Supplementary materials (figures, tables, graphical abstract, slides, videos, Chinese translated version, and updated materials) are available via the article DOI or on iMeta Science (http://www.imeta.science).

## References

[1] McIntyre, H. David , Patrick Catalano , Cuilin Zhang , Gernot Desoye , Elisabeth R. Mathiesen , and Peter Damm . 2019. “Gestational Diabetes Mellitus.” Nature Reviews Disease Primers 5: 47. 10.1038/s41572-019-0098-8 31296866

[2] Venkatesh, Kartik K. , Courtney D. Lynch , Camille E. Powe , Maged M. Costantine , Stephen F. Thung , Steven G. Gabbe , William A. Grobman , and Mark B. Landon . 2022. “Risk of Adverse Pregnancy Outcomes Among Pregnant Individuals With Gestational Diabetes by Race and Ethnicity in the United States, 2014–2020.” JAMA 327: 1356–1367. 10.1001/jama.2022.3189 35412565 PMC9006108

[3] Vounzoulaki, Elpida , Kamlesh Khunti , Sophia C. Abner , Bee K. Tan , Melanie J. Davies , and Clare L. Gillies . 2020. “Progression to Type 2 Diabetes in Women With a Known History of Gestational Diabetes: Systematic Review and Meta‐Analysis.” BMJ 369: m1361. 10.1136/bmj.m1361 32404325 PMC7218708

[4] Ye, Wenrui , Cong Luo , Jing Huang , Chenglong Li , Zhixiong Liu , and Fangkun Liu . 2022. “Gestational Diabetes Mellitus and Adverse Pregnancy Outcomes: Systematic Review and Meta‐Analysis.” BMJ 377: e067946. 10.1136/bmj-2021-067946 35613728 PMC9131781

[5] Davidson, Karina W. , Michael J. Barry , Carol M. Mangione , Michael Cabana , Aaron B. Caughey , Esa M. Davis , Katrina E. Donahue , et al. 2021. “Screening for Gestational Diabetes: US Preventive Services Task Force Recommendation Statement.” JAMA 326: 531–538. 10.1001/jama.2021.11922 34374716

[6] Sweeting, Arianne , Jencia Wong , Helen R. Murphy , and Glynis P. Ross . 2022. “A Clinical Update on Gestational Diabetes Mellitus.” Endocrine Reviews 43: 763–793. 10.1210/endrev/bnac003 35041752 PMC9512153

[7] Wang, Xing , Hongli Liu , Yifan Li , Shuai Huang , Lan Zhang , Chiying Cao , Philip N. Baker , et al. 2020. “Altered Gut Bacterial and Metabolic Signatures and Their Interaction in Gestational Diabetes Mellitus.” Gut Microbes 12: 1840765. 10.1080/19490976.2020.1840765 33222612 PMC7714515

[8] Wang, Yi , Yichao Huang , Ping Wu , Yi Ye , Fengjiang Sun , Xue Yang , Qi Lu , et al. 2021. “Plasma Lipidomics in Early Pregnancy and Risk of Gestational Diabetes Mellitus: A Prospective Nested Case‐Control Study in Chinese Women.” American Journal of Clinical Nutrition 114: 1763–1773. 10.1093/ajcn/nqab242 34477820

[9] Wang, Jinfeng , Jiayong Zheng , Wenyu Shi , Nan Du , Xiaomin Xu , Yanming Zhang , Peifeng Ji , et al. 2018. “Dysbiosis of Maternal and Neonatal Microbiota Associated With Gestational Diabetes Mellitus.” Gut 67: 1614–1625. 10.1136/gutjnl-2018-315988 29760169 PMC6109274

[10] Ye, Dewei , Jiating Huang , Jiaming Wu , Kang Xie , Xiang Gao , Kaixuan Yan , Pengfei Zhang , et al. 2023. “Integrative Metagenomic and Metabolomic Analyses Reveal Gut Microbiota‐Derived Multiple Hits Connected to Development of Gestational Diabetes Mellitus in Humans.” Gut Microbes 15: 2154552. 10.1080/19490976.2022.2154552 36550785 PMC9794004

[11] Brown, Eric M. , Jon Clardy , and Ramnik J. Xavier . 2023. “Gut Microbiome Lipid Metabolism and Its Impact on Host Physiology.” Cell Host & Microbe 31: 173–186. 10.1016/j.chom.2023.01.009 36758518 PMC10124142

[12] Li, Chenhao , Martin Stražar , Ahmed M. T. Mohamed , Julian A. Pacheco , Rebecca L. Walker , Tina Lebar , Shijie Zhao , et al. 2024. “Gut Microbiome and Metabolome Profiling in Framingham Heart Study Reveals Cholesterol‐Metabolizing Bacteria.” Cell 187: 1834–1852.e19. 10.1016/j.cell.2024.03.014 38569543 PMC11071153

[13] Wahlström, Annika , Sama I. Sayin , Hanns‐Ulrich Marschall , and Fredrik Bäckhed . 2016. “Intestinal Crosstalk Between Bile Acids and Microbiota and Its Impact on Host Metabolism.” Cell Metabolism 24: 41–50. 10.1016/j.cmet.2016.05.005 27320064

[14] Schoeler, Marc , and Robert Caesar . 2019. “Dietary Lipids, Gut Microbiota and Lipid Metabolism.” Reviews in Endocrine and Metabolic Disorders 20: 461–472. 10.1007/s11154-019-09512-0 31707624 PMC6938793

[15] Johnson, Elizabeth L. , Stacey L. Heaver , Jillian L. Waters , Benjamin I. Kim , Alexis Bretin , Andrew L. Goodman , Andrew T. Gewirtz , Tilla S. Worgall , and Ruth E. Ley . 2020. “Sphingolipids Produced by Gut Bacteria Enter Host Metabolic Pathways Impacting Ceramide Levels.” Nature Communications 11: 2471. 10.1038/s41467-020-16274-w PMC7235224 32424203

[16] Mei, Zhendong , Fenglei Wang , Amrisha Bhosle , Danyue Dong , Raaj Mehta , Andrew Ghazi , Yancong Zhang , et al. 2024. “Strain‐Specific Gut Microbial Signatures in Type 2 Diabetes Identified in a Cross‐Cohort Analysis of 8,117 Metagenomes.” Nature Medicine 30: 2265–2276. 10.1038/s41591-024-03067-7 PMC11620793 38918632

[17] Ze, Xiaolei , Sylvia H. Duncan , Petra Louis , and Harry J. Flint . 2012. “ *Ruminococcus bromii* is a Keystone Species for the Degradation of Resistant Starch in the Human Colon.” The ISME Journal 6: 1535–1543. 10.1038/ismej.2012.4 22343308 PMC3400402

[18] Hosomi, Koji , Mayu Saito , Jonguk Park , Haruka Murakami , Naoko Shibata , Masahiro Ando , Takahiro Nagatake , et al. 2022. “Oral Administration of *Blautia wexlerae* Ameliorates Obesity and Type 2 Diabetes via Metabolic Remodeling of the Gut Microbiota.” Nature Communications 13: 4477. 10.1038/s41467-022-32015-7 PMC9388534 35982037

[19] Qin, Junjie , Yingrui Li , Zhiming Cai , Shenghui Li , Jianfeng Zhu , Fan Zhang , Suisha Liang , et al. 2012. “A Metagenome‐Wide Association Study of Gut Microbiota in Type 2 Diabetes.” Nature 490: 55–60. 10.1038/nature11450 23023125

[20] Dicks, Leon M. T . 2024. “How Important Are Fatty Acids in Human Health and Can They Be Used in Treating Diseases?” Gut Microbes 16: 2420765. 10.1080/19490976.2024.2420765 39462280 PMC11520540

[21] Liebisch, Gerhard , Eoin Fahy , Junken Aoki , Edward A. Dennis , Thierry Durand , Christer S. Ejsing , Maria Fedorova , et al. 2020. “Update on LIPID MAPS Classification, Nomenclature, and Shorthand Notation for MS‐Derived Lipid Structures.” Journal of Lipid Research 61: 1539–1555. 10.1194/jlr.S120001025 33037133 PMC7707175

[22] Kuo, Andrew , and Timothy Hla . 2024. “Regulation of Cellular and Systemic Sphingolipid Homeostasis.” Nature Reviews Molecular Cell Biology 25: 802–821. 10.1038/s41580-024-00742-y 38890457 PMC12034107

[23] Mokkala, Kati , Niklas Paulin , Noora Houttu , Ella Koivuniemi , Outi Pellonperä , Sofia Khan , Sami Pietilä , et al. 2021. “Metagenomics Analysis of Gut Microbiota in Response to Diet Intervention and Gestational Diabetes in Overweight and Obese Women: A Randomised, Double‐Blind, Placebo‐Controlled Clinical Trial.” Gut 70: 309–318. 10.1136/gutjnl-2020-321643 32839200

[24] Pinto, Yishay , Sigal Frishman , Sondra Turjeman , Adi Eshel , Meital Nuriel‐Ohayon , Oshrit Shtossel , Oren Ziv , et al. 2023. “Gestational Diabetes Is Driven by Microbiota‐Induced Inflammation Months Before Diagnosis.” Gut 72: 918–928. 10.1136/gutjnl-2022-328406 36627187 PMC10086485

[25] Lyu, Xuejing , Shaona Wang , Jiaxin Zhong , Lingzhu Cai , Yanhui Zheng , Ying Zhou , Ying Zhou , Qionghua Chen , and Qiyuan Li . 2023. “Gut Microbiome Interacts With Pregnancy Hormone Metabolites in Gestational Diabetes Mellitus.” Frontiers in Microbiology 14: 1175065. 10.3389/fmicb.2023.1175065 37492251 PMC10364628

[26] Sun, Zhonghan , Xiong‐Fei Pan , Xiao Li , Limiao Jiang , Ping Hu , Yi Wang , Yi Ye , et al. 2023. “The Gut Microbiome Dynamically Associates With Host Glucose Metabolism Throughout Pregnancy: Longitudinal Findings From a Matched Case‐Control Study of Gestational Diabetes Mellitus.” Advanced Science 10: e2205289. 10.1002/advs.202205289 36683149 PMC10074094

[27] Muniyappa, Ranganath . 2024. “Vascular Insulin Resistance and Free Fatty Acids: The Micro‐Macro Circulation Nexus.” The Journal of Clinical Endocrinology & Metabolism 109: e1671–e1672. 10.1210/clinem/dgae013 38181432

[28] Samuel, Varman T. , Kitt Falk Petersen , and Gerald I. Shulman . 2010. “Lipid‐Induced Insulin Resistance: Unravelling the Mechanism.” The Lancet 375: 2267–2277. 10.1016/s0140-6736(10)60408-4 PMC2995547 20609972

[29] Petersen, Max C. , and Gerald I. Shulman . 2017. “Roles of Diacylglycerols and Ceramides in Hepatic Insulin Resistance.” Trends in Pharmacological Sciences 38: 649–665. 10.1016/j.tips.2017.04.004 28551355 PMC5499157

[30] Birkenfeld, Andreas L. , and Gerald I. Shulman . 2014. “Nonalcoholic Fatty Liver Disease, Hepatic Insulin Resistance, and Type 2 Diabetes.” Hepatology 59: 713–723. 10.1002/hep.26672 23929732 PMC3946772

[31] Hornburg, Daniel , Si Wu , Mahdi Moqri , Xin Zhou , Kevin Contrepois , Nasim Bararpour , Gavin M. Traber , et al. 2023. “Dynamic Lipidome Alterations Associated With Human Health, Disease and Ageing.” Nature Metabolism 5: 1578–1594. 10.1038/s42255-023-00880-1 PMC10513930 37697054

[32] Apostolopoulou, Maria , Ruth Gordillo , Chrysi Koliaki , Sofia Gancheva , Tomas Jelenik , Elisabetta De Filippo , Christian Herder , et al. 2018. “Specific Hepatic Sphingolipids Relate to Insulin Resistance, Oxidative Stress, and Inflammation in Nonalcoholic Steatohepatitis.” Diabetes Care 41: 1235–1243. 10.2337/dc17-1318 29602794

[33] Chaurasia, Bhagirath , Trevor S. Tippetts , Rafael Mayoral Monibas , Jinqi Liu , Ying Li , Liping Wang , Joseph L. Wilkerson , et al. 2019. “Targeting a Ceramide Double Bond Improves Insulin Resistance and Hepatic Steatosis.” Science 365: 386–392. 10.1126/science.aav3722 31273070 PMC6787918

[34] Brown, Eric M. , Xiaobo Ke , Daniel Hitchcock , Sarah Jeanfavre , Julian Avila‐Pacheco , Toru Nakata , Timothy D. Arthur , et al. 2019. “ *Bacteroides*‐Derived Sphingolipids Are Critical for Maintaining Intestinal Homeostasis and Symbiosis.” Cell Host & Microbe 25: 668–680.e667. 10.1016/j.chom.2019.04.002 31071294 PMC6544385

[35] Luo, Jing , and Yulan Wang . 2025. “Precision Dietary Intervention: Gut Microbiome and Meta‐Metabolome as Functional Readouts.” Phenomics 5: 23–50. 10.1007/s43657-024-00193-7 40313608 PMC12040796

[36] International Association of Diabetes and Pregnancy Study Groups Consensus Panel , Boyd E. Metzger , Steven G. Gabbe , Bengt Persson , Thomas A. Buchanan , Patrick A. Catalano , Peter Damm , Alan R. Dyer , et al. 2010. “International Association of Diabetes and Pregnancy Study Groups Recommendations on the Diagnosis and Classification of Hyperglycemia in Pregnancy.” Diabetes Care 33: e98. 10.2337/dc10-0719 20190296 PMC2827530

[37] Wallace, Tara M. , Jonathan C. Levy , and David R. Matthews . 2004. “Use and Abuse of HOMA Modeling.” Diabetes Care 27: 1487–1495. 10.2337/diacare.27.6.1487 15161807

[38] Pan, Xiong‐Fei , Yichao Huang , Xinping Li , Yi Wang , Yi Ye , Huan Chen , Matti Marklund , et al. 2021. “Circulating Fatty Acids and Risk of Gestational Diabetes Mellitus: Prospective Analyses in China.” European Journal of Endocrinology 185: 87–97. 10.1530/eje-21-0118 33914701

[39] Wang, Yetong , Ruyi Zhang , Yanni Pu , Danqi Wang , Yanren Wang , Xuemei Wu , Yujie Pan , et al. 2023. “Sample Collection, DNA Extraction, and Library Construction Protocols of the Human Microbiome Studies in the International Human Phenome Project.” Phenomics 3: 300–308. 10.1007/s43657-023-00097-y 37325707 PMC10260709

[40] Pu, Yanni , Xiaofeng Zhou , Hao Cai , Tao Lou , Chenglin Liu , Mengmeng Kong , Zhonghan Sun , et al. 2025. “Impact of DNA Extraction Methods on Gut Microbiome Profiles: A Comparative Metagenomic Study.” Phenomics 5: 76–90. 10.1007/s43657-025-00232-x 40313603 PMC12040788

[41] Bolger, Anthony M. , Marc Lohse , and Bjoern Usadel . 2014. “Trimmomatic: A Flexible Trimmer for Illumina Sequence Data.” Bioinformatics 30: 2114–2120. 10.1093/bioinformatics/btu170 24695404 PMC4103590

[42] Langmead, Ben , and Steven L. Salzberg . 2012. “Fast Gapped‐Read Alignment with Bowtie 2.” Nature Methods 9: 357–359. 10.1038/nmeth.1923 22388286 PMC3322381

[43] Beghini, Francesco , Lauren J. McIver , Aitor Blanco‐Míguez , Leonard Dubois , Francesco Asnicar , Sagun Maharjan , Ana Mailyan , et al. 2021. “Integrating Taxonomic, Functional, and Strain‐Level Profiling of Diverse Microbial Communities with Biobakery 3.” eLife 10: e65088. 10.7554/eLife.65088 33944776 PMC8096432

[44] Caspi, Ron , Richard Billington , Ingrid M. Keseler , Anamika Kothari , Markus Krummenacker , Peter E. Midford , Wai Kit Ong , et al. 2020. “The MetaCyc Database of Metabolic Pathways and Enzymes—A 2019 Update.” Nucleic Acids Research 48: D445–D453. 10.1093/nar/gkz862 31586394 PMC6943030

[45] Alshehry, Zahir , Christopher Barlow , Jacquelyn Weir , Youping Zhou , Malcolm McConville , and Peter Meikle . 2015. “An Efficient Single Phase Method for the Extraction of Plasma Lipids.” Metabolites 5: 389–403. 10.3390/metabo5020389 26090945 PMC4495379

[46] Züllig, Thomas , Martin Trötzmüller , and Harald C. Köfeler . 2020. “Lipidomics From Sample Preparation to Data Analysis: A Primer.” Analytical and Bioanalytical Chemistry 412: 2191–2209. 10.1007/s00216-019-02241-y 31820027 PMC7118050

[47] Wang, Yi , Ping Wu , Yichao Huang , Yi Ye , Xue Yang , Fengjiang Sun , Yi‐Xiang Ye , et al. 2022. “BMI and Lipidomic Biomarkers With Risk of Gestational Diabetes in Pregnant Women.” Obesity 30: 2044–2054. 10.1002/oby.23517 36046944

[48] Mallick, Himel , Ali Rahnavard , Lauren J. McIver , Siyuan Ma , Yancong Zhang , Long H. Nguyen , Timothy L. Tickle , et al. 2021. “Multivariable Association Discovery in Population‐Scale Meta‐Omics Studies.” PLOS Computational Biology 17: e1009442. 10.1371/journal.pcbi.1009442 34784344 PMC8714082

[49] Savvidou, Makrina , Scott M. Nelson , Mahlatse Makgoba , Claudia‐Martina Messow , Naveed Sattar , and Kypros Nicolaides . 2010. “First‐Trimester Prediction of Gestational Diabetes Mellitus: Examining the Potential of Combining Maternal Characteristics and Laboratory Measures.” Diabetes 59: 3017–3022. 10.2337/db10-0688 20876721 PMC2992761

[50] Lai, Senying , Yan Yan , Yanni Pu , Shuchun Lin , Jian‐Ge Qiu , Bing‐Hua Jiang , Marisa Isabell Keller , et al. 2023. “Enterotypes of the Human Gut Mycobiome.” Microbiome 11: 179. 10.1186/s40168-023-01586-y 37563687 PMC10416509

[51] Zhen, Jianxin , Yuqin Gu , Piao Wang , Weihong Wang , Shengzhe Bian , Shujia Huang , Hui Liang , et al. 2024. “Genome‐Wide Association and Mendelian Randomisation Analysis Among 30,699 Chinese Pregnant Women Identifies Novel Genetic and Molecular Risk Factors for Gestational Diabetes and Glycaemic Traits.” Diabetologia 67: 703–713. 10.1007/s00125-023-06065-5 38372780 PMC10904416

