# Supplementary material for: A gut microbiome‐lipid axis in early pregnancy is associated with metabolic dysregulation and diabetes risk
Source: Imeta. 2026 Aug 10;5(4):e70166. doi: 10.1002/imt2.70166 (PMC13455838; doi:10.1002/imt2.70166)
Supplement: Supplementary file 1 [file IMT2-5-e70166-s002.docx]

**Supporting information to**

**A gut microbiome-lipid axis in early pregnancy is associated with metabolic dysregulation and diabetes risk**

**Running title**: Gut microbiome-lipidome axis and GDM risk

Zhonghan Sun^1#^, Chang He^1#^, Xiaonan Ma^1#^, Ping Wu^2#^, Tianlei Wang^3, 4, 5, 6^, Jiaying Yuan^7^, Yanni Pu^1^, Xiaofeng Zhou^1^, Zhendong Mei^8^, Huiling Song^1^, Yetong Wang^1^, Haiyan Yue^1^, Yuanqing Fu^9^, Jusheng Zheng^9^, An Pan^2^, Da Chen^10*^, Shangyu Hong^11*^, Xiong-Fei Pan^3, 4, 5, 6*^, Yan Zheng^1, 12, 13*^

^1^ State Key Laboratory of Genetic Engineering, Human Phenome Institute, School of Life Sciences, Zhongshan Hospital, Fudan University, Shanghai 200433, China.

^2^ Department of Epidemiology and Biostatistics, Ministry of Education Key Laboratory of Environment and Health, School of Public Health, Tongji Medical College, Huazhong University of Science and Technology, Wuhan 430030, China.

^3^ Laboratory of Epidemiology and Population Health & Children's Medicine Key Laboratory of Sichuan Province, West China Institute of Women and Children’s Health, West China Second University Hospital, Sichuan University, Chengdu 610041, China.

^4^ Department of Obstetrics and Gynecology, West China Second University Hospital, Sichuan University, Chengdu 610041, China.

^5^ Key Laboratory of Birth Defects and Related Diseases of Women and Children (Sichuan University), Ministry of Education, Chengdu 610041, China.

^6^ Shuangliu Institute of Women's and Children's Health, Shuangliu Maternal and Child Health Hospital, Chengdu 610200, China.

^7^ Department of Science and Education, Shuangliu Maternal and Child Health Hospital, Chengdu 610200, China.

^8^ Department of Medicine, Brigham and Women's Hospital and Harvard Medical School, Boston 02115, USA.

^9^ Affiliated Hangzhou First People’s Hospital, School of Medicine, Westlake University, Hangzhou 310024, China.

^10^ School of Environment, Guangdong Key Laboratory of Environmental Pollution and Health, Jinan University, Guangzhou 510632, China.

^11^ Department of Endocrinology, Shanghai Pudong Hospital, Fudan University Pudong Medical Center, State Key Laboratory of Genetics and Development of Complex Phenotypes, School of Life Sciences, Fudan University, Shanghai 201209, China.

^12^ Ministry of Education Key Laboratory of Public Health Safety, School of Public Health, Institute of Nutrition, Fudan University, Shanghai 200032‌, China.

^13^ National Clinical Research Center for Aging and Medicine, Huashan Hospital, Fudan University, Shanghai 200040, China.

^#^ These authors contributed equally: Zhonghan Sun, Chang He, Xiaonan Ma, Ping Wu

*Correspondence: [yan_zheng@fudan.edu.cn](mailto:yan_zheng@fudan.edu.cn) (Yan Zheng); [pxiongfei@scu.edu.cn](mailto:pxiongfei@scu.edu.cn) (Xiong-Fei Pan); [shangyu_hong@fudan.edu.cn (Shangyu Hong);](mailto:shangyu_hong@fudan.edu.cn%20(Shangyu%20Hong);)[dachen@jnu.edu.cn](mailto:dachen@jnu.edu.cn) (Da Chen)

**Figure S1** **The association between microbial species and clinical measurements related to glucose and lipid metabolism.** The colors of heatmap indicated the linear regression coefficient calculated by MaAsLin2, adjusting for potential covariates including maternal age, gestational age, education, parity, family history of diabetes, history of PCOS, pre-pregnancy BMI, cigarette smoking, alcohol drinking, physical activity, and use of antibiotics. GDM, gestational diabetes mellitus. OGTT, oral glucose tolerance test. FPG, fasting plasma glucose. BMI, body mass index. HbA1c, hemoglobin A1c. SBP, systolic blood pressure. HOMA-IR, homeostatic model assessment for insulin resistance. GWG, gestational weight gain. TG, triglycerides. LDL-C, low-density lipoprotein cholesterol. Apo, apolipoprotein. * FDR < 0.25; ** FDR<0.10, *** FDR <0.05.

**Figure S2 The proportions of enzymes in microbial functional potentials of fatty acids metabolism encoded by specific species.**

**Figure S3** **The associations between lipids with different numbers of acyl chain carbons and GDM risk.** Each dot represented a lipid metabolite, with the color representing its classification. All the showed lipids were significantly associated with GDM risk after adjusting for maternal age, gestational age, education, parity, family history of diabetes, history of PCOS, pre-pregnancy BMI, cigarette smoking, alcohol drinking, physical activity, and use of antibiotics.

**Figure S4** **Procrustes analysis of gut microbiome composition and plasma lipidome profile.** Procrustes analysis plot illustrating the similarity between the gut microbiome composition and the plasma lipidome profile. Each arrow represents the mapping of a sample from the microbiome space to the lipidome space. The tight clustering of points around the origin and the short length of the arrows indicate a strong similarity between the two datasets. The statistical significance was assessed using Monte Carlo simulation.

**Figure S5 The trans-omics associations between GDM-related species and lipid metabolites.** The colors of heatmap indicated the linear regression coefficient calculated by MaAsLin2, adjusting for potential covariates including maternal age, gestational age, education, parity, family history of diabetes, history of PCOS, pre-pregnancy BMI, cigarette smoking, alcohol drinking, physical activity, and use of antibiotics. GDM, gestational diabetes mellitus. *, FDR < 0.25; **, FDR<0.10; ***, FDR <0.05.

**Figure S6 The trans-omics associations between GDM-related microbial metabolic potentials and lipid metabolites.** The colors of heatmap indicated the linear regression coefficient calculated by MaAsLin2, adjusting for potential covariates including maternal age, gestational age, education, parity, family history of diabetes, pre-pregnancy BMI, cigarette smoking, alcohol drinking, physical activity, and use of antibiotics. GDM, gestational diabetes mellitus. *, FDR < 0.25; **, FDR<0.10; ***, FDR<0.05.

**Figure S7 Circulating lipid parameters following in vivo intervention.** Serum levels of total cholesterol (TC), triglycerides (TG), high-density lipoprotein (HDL), and low-density lipoprotein (LDL) were measured in mice treated with LacCer 24:1 or vehicle (DMSO), as well as in mice colonized with Ruminococcus bicirculans or PBS control. Data are presented as mean ± SEM with individual data points. Statistical comparisons were performed between intervention and corresponding control groups. Most lipid parameters remained unchanged across groups, with triglycerides showing significant differences. ns, not significant; *, *P* < 0.05; **, *P* < 0. 001.

**Figure S8 Body weight trajectories during the in vivo intervention experiment.** (A) Body weight changes in mice treated with LacCer 24:1 or vehicle (DMSO). (B) Body weight changes in mice colonized with *Ruminococcus bicirculans* or PBS control. Data are presented as mean ± SEM with individual data points. Group differences were assessed at each time point using two-sided unpaired Student’s t-tests, followed by Benjamini–Hochberg correction for multiple comparisons. No significant differences were observed between groups at any time point. ns, not significant; FDR > 0.05.
